# Neferine Attenuates Acute Kidney Injury by Inhibiting NF-κB Signaling and Upregulating Klotho Expression

**DOI:** 10.3389/fphar.2019.01197

**Published:** 2019-10-15

**Authors:** Huihui Li, Wenhang Chen, Yusa Chen, Qiaoling Zhou, Ping Xiao, Rong Tang, Jing Xue

**Affiliations:** ^1^Department of Nephrology, Xiangya Hospital, Central South University, Changsha, China; ^2^Institute of Hospital Administration, Xiangya Hospital, Central South University, Changsha, China; ^3^Department of Scientific Research, Xiangya Hospital, Central South University, Changsha, China

**Keywords:** acute kidney injury (AKI), neferine, NF-κB, Klotho, apoptosis, inflammation

## Abstract

**Purpose:** Morbidity associated with and mortality from acute kidney injury (AKI) is gradually increasing, and no efficient drug is available. We explored whether neferine, a bisbenzylisoquinoline alkaloid, attenuated AKI, and the possible mechanisms in play *in vivo* and *in vitro*.

**Methods:** We induced AKI using ischemia-reperfusion (I/R) or lipopolysaccharide (LPS) *in vivo*. C57 BL/6 male mice were randomized into two groups each containing four subgroups: control, neferine, I/R or LPS, and I/R or LPS + neferine. Mice were sacrificed 24 h after AKI induction and kidneys and sera were collected. NRK-52E cells were exposed to hypoxia/reoxygenation (H/R) or LPS *in vitro*.

**Results:** Neferine pretreatment significantly alleviated kidney functional loss and pathological damage. In the AKI mouse models induced by I/R or LPS, neferine inhibited the infiltration of inflammatory cells, including granulocytes and macrophages. Both *in vivo* and *in vitro*, neferine attenuated apoptosis, suppressed inflammatory cytokine production, decreased degradation of IκB-α, and inhibited nuclear translocation of NF-κB. Furthermore, it also upregulated Klotho expression in AKI.

**Conclusion:** Neferine mitigated renal injury in AKI models, perhaps by suppressing the activation of NF-κB and upregulating the expression of Klotho.

## Introduction

Acute kidney injury (AKI) is a heterogeneous group of disorders manifested by increased levels of serum creatinine (Scr) and/or oliguria. AKI is becoming more prevalent worldwide, associated with high mortality and morbidity (Ali et al., 2007; Bellomo et al., 2012; Wen et al., 2013). A systematic review of data from 2004 to 2012 found that the pooled adult AKI rate was 21.6% [95% confidence interval (CI) 19.3 to 24.1%], while the pooled mortality of AKI patients was 23.9% (95% CI 22.1 to 25.7%) (Susantitaphong et al., 2013). The multinational AKI–epidemiologic prospective investigation found that AKI developed in over half of all intensive care unit patients (Hoste et al., 2015). AKI was associated with prolonged hospitalization, more resource utilization, and an unfavorable prognosis. AKI prevention and treatment are thus urgent. Generally, AKI develops after the contraction of another acute or chronic disease such as a critical illness, sepsis, circulatory shock, or complications associated with medication (Levey and James, 2017). When the kidneys are exposed to risk factors, various cellular reactions causing tissue injury commence. Inflammation plays a key role in AKI emergence, exacerbation, and prognosis (Akcay et al., 2009; Dellepiane et al., 2016; Coelho et al., 2018); it involves the release of inflammatory mediators by endothelial and tubular cells, infiltration of inflammatory cells, and the damaging effects of toxic molecules on renal tubules. Nuclear factor kappa B (NF-κB) activation has been recorded both *in vitro* and *in vivo* models of AKI. Research increasingly suggests that NF-κB plays a pivotal role in AKI-associated inflammation and other cellular events (Guijarro and Egido, 2001; Sanz et al., 2010; Panah et al., 2018). Renal tubular epithelial cells stimulated by lipopolysaccharide (LPS) produce inflammatory mediators, free radicals, and other disruptive agents; express various proinflammatory cytokines after NF-κB activation; and, ultimately, contribute greatly to renal dysfunction. The level of Klotho, a single-pass transmembrane protein, correlates with the levels of aging suppressors (Kuro-O et al., 1997) and the extent of calcium/phosphorus metabolism (Huang and Moe, 2011). Klotho is synthesized principally by the distal renal tubules. AKI is associated with greatly reduced Klotho production, in turn aggravating kidney damage (Hu et al., 2010; Hu et al., 2012).

**Figure 1:**
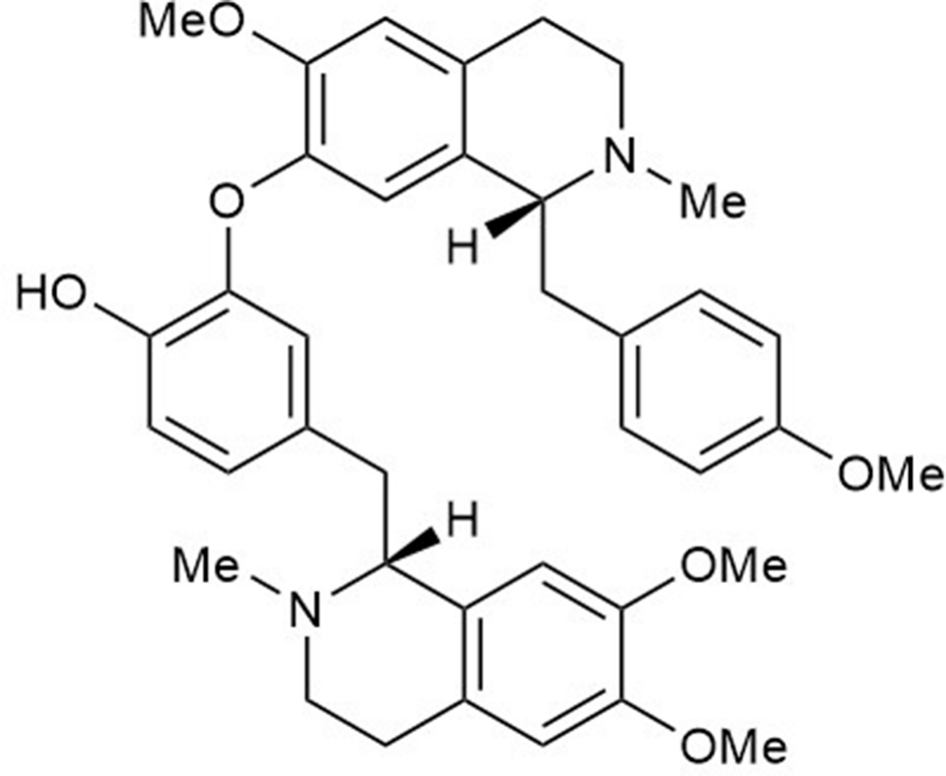
The chemical structure of neferine (C_38_H_44_N_2_O_6_).

Neferine (**Figure 1**), a bisbenzylisoquinoline alkaloid from the seed embryo of *Nelumbo nucifera*, is widely used in traditional Chinese medicine to treat nervous exhaustion, bacillosis, helminthiasis, and cardiovascular and pulmonary diseases (Sharma et al., 2017; Marthandam Asokan et al., 2018). Neferine exhibits anti-cancer properties (Qin et al., 2011; Deng et al., 2017), relaxes smooth muscles; inhibits platelet aggregation and arrhythmia; retards the development of atherosclerosis (Zheng et al., 2014), diabetes, and Alzheimer’s disease; and reduces scarring and cisplatin nephrotoxicity (Li et al., 2017), and the like. Neferine attenuates the inflammatory response and fibrosis by inhibiting cytokine production and NF-κB activation both *in vivo* and *in vitro* (Zhao et al., 2010; Baskaran et al., 2016; Baskaran et al., 2017; Priya et al., 2017; Guolan et al., 2018). Thus, we speculated that neferine might protect the kidney from acute injury. We established two AKI models to explore this topic.

## Materials and Methods

### Animals

C57BL/6 male mice with 10 weeks of age (20–25 g in weight) were purchased from the Department of Laboratory Animals of Central South University and were individually housed at a standard temperature (22 ± 2°C) and humidity (50–60%) under a 12-h/12-h photoperiod. The study was approved by the Institutional Animal Care and Use Committee of Central South University. All animal experimental procedures met the criteria of the National Institutes of Health (NIH) Guide for the Care and Use of Laboratory Animals.

AKI was induced by ischemia-reperfusion (I/R) or LPS injection as described previously (Savransky et al., 2006; Cao et al., 2008). The mice were randomized into two major groups and each group then divided into four subgroups (n = 6 mice per subgroup): (1) control, (2) neferine (Purechem-Standard Co. Ltd., Chengdu, China), (3) I/R or LPS, and (4) I/R or LPS + neferine. The I/R model was established by clipping both kidney pedicles (using microarterial clamps; model FST 18055-04: Fine Science Tools Inc., Foster City, CA, USA) for 30 min. LPS-induced AKI was generated *via* intraperitoneal injection of 10 mg/kg LPS. Neferine was administrated *via* intraperitoneal injection for 3 days prior to model establishment (20 mg/kg/d) (Huang et al., 2007); mice were sacrificed 24 h later. Normal saline was used instead of neferine in control and model groups. Anesthesia was induced by an intraperitoneal injection of pentobarbital (0.7 mg/kg) prior to surgery. Kidneys and sera were stored at −80°C prior to analysis.

### Histology

Kidneys were fixed in 4% (v/v) paraformaldehyde for 48 h at room temperature. Paraffin-embedded sections (4 µm thick) were stained with hematoxylin and eosin and periodic acid–Schiff. The extent of tubular injury was scored as the percentage of damaged tubules in the outer medulla (Savransky et al., 2006). All scoring was performed by a renal pathologist (blinded to mouse subgroup) under the high-power field of a light microscope. Ten random non-overlapping fields were examined for each sample.

### Serum Biochemistry and Enzyme-Linked Immunosorbent Assay (ELISA)

Scr and blood urine nitrogen (BUN) levels were measured using a Hitachi model 7180 automatic analyzer (Hitachi Ltd., Tokyo, Japan). Serum levels of neutrophil gelatinase-associated lipocalin (NGAL) were measured with the aid of an ELISA kit following the manufacturer’s instructions (Abcam, Cambridge, UK).

### Immunohistochemical Staining

Kidney sections were sequentially deparaffinized, rehydrated, and subjected to antigen retrieval and inactivation of endogenous enzyme activity. After blocking in 5% (w/v) bovine serum album (BSA) for 30 min, tissues were incubated with a primary antibody targeting Ly6G (Servicebio, Wuhan, China; 1:200), and F4/80 (Servicebio; 1:200) and Klotho (Abcam; 1:200) at 4°C overnight. Biotinylated secondary antibodies (ZsBio, Beijing, China) were then added; the samples were incubated at room temperature for 30 min and analyzed using a biotin-streptavidin horseradish peroxidase (HRP) detection system.

### Cell Culture

NRK-52E cells (American Type Culture Collection, Rockville, MD, USA) were cultured in high-glucose Dulbecco’s modified Eagle’s medium (DMEM) containing 10% fetal bovine serum (FBS) at 37°C in a humidified incubator with an atmosphere containing 5% CO2. Two cell-based models of AKI were established—hypoxia/reoxygenation (H/R)– and LPS (5 µg/ml, Sigma)-mediated cell injuries. Cells were pretreated with the indicated concentrations of neferine for 2 h, followed by H/R or administration of LPS. For H/R, the cells were cultured in glucose- and FBS-free DMEM containing antimycin A (100 nM, Abcam) for 2 h to imitate hypoxia. The cells were subsequently cultured in normal medium until being harvested.

### Cell Counting Kit-8 Assay

A Cell Counting Kit-8 (CCK8) (Selleck, Shanghai, China) was used to assay cell proliferation. NRK-52E cells were seeded in 96-well plates at a density of 4 × 10^3^/well and incubated overnight. When the cells reached 50% confluence, neferine was added at the indicated concentrations (1, 2, 4, 8, 10, 20, 40, 100μM), and the cells were incubated for 24 h. Next, 10 ml of CCK-8 reagent was added to each well, and the plate was incubated at 37°C for 2 h. Finally, the absorbance at 450 nm was measured. Each experiment was conducted independently three times.

### Flow Cytometry

An Annexin V-Fluorescein Isothiocyanate (FITC)/Propidium Iodide (PI) Apoptosis Detection Kit (BD Pharmingen, CA, USA) was used to assay the rate of apoptosis of cultured cells. Cells were collected by trypsinization 12 h after H/R. After washing twice with cold PBS, the cells were resuspended in 300 ml of 1× binding buffer and stained with 5 ml of Annexin V-FITC plus 5 ml of PI for 15 min in the dark. Fluorescence signals were detected using a FACScan Flow Cytometer (BD Biosciences, CA, USA). Each experiment was conducted independently three times.

### Quantitative Reverse Transcription-PCR

Total RNAs were isolated from kidney tissue NRK-52E cells exposed to the TransZol Up reagent (TransGen Biotech, Beijing, China) and reverse-transcribed into complementary DNA using the EasyScript One-Step gDNA Removal and cDNA Synthesis SuperMix system (TransGen Biotech). Quantitative PCR employed Hiff qPCR SYBR Green Master Mix (Low ROX Plus) (Yeasen, Shanghai, China) and the following primers: M-Klotho, forward 5´-TTTGCCCTATTTCACCGAAG-3,´ reverse 5´-CCTGACT​GGGAGAGTTGAGC-3´; M-tumor necrosis factor-α (TNF-α), forward 5´-TATGGCTCAGGGTCCAACTC-3,´ reverse 5´-CTCCCTTTGCAGAACTCAGG-3´; M-interleukin-6 (IL-6), forward5´-AGTTGCCTTCTTGGGACTGA-3,´ reverse 5´-TCCACGATTTCCCAGAGAAC-3´; M-cyclooxygenase 2 (COX2), forward 5´-TGCAGAATTGAAAGCCCTCT-3,´ reverse 5´-GCTCGGCTTCCAGTATTGAG-3´; and M-β-actin, forward 5´-CGTTGACATCCGTAAAGACC-3,´ reverse 5´-AACAGTCCGCCTAGAAGCAC-3´; R-Klotho, forward 5´-AGCTGCTTGTGTTGTGATGC-3,´ reverse 5´-TACGGGGGTGCTGTAGAAAC-3´; R-TNF-α, forward 5´-AACTCCCAGAAAAGCAAGCA-3,´ reverse 5´-CGAGCAGGAATGAGAAGAGG-3´; and R-β-actin, forward 5´-CACCCGCGAGTACAACCTTC-3,´ reverse 5´-CCCATACCCACCATCACACC-3.´

### Western Blotting

Total protein was extracted from mouse kidneys and cultured cells using RIPA Lysis Buffer (Beyotime Biotechnology, Nanjing, China). Nucleoproteins were extracted using a commercial kit (Beyotime Biotechnology). Samples (20–40 µg of protein/lane) were separated on 12% (w/v) sodium dodecyl sulfate-polyacrylamide gels; the proteins were electrotransferred to 0.22-µm pore-sized polyvinylidene fluoride membranes (Millipore Sigma, Burlington, MA, USA) and immunoblotted with primary antibodies against Klotho (Abcam; 1:1,000; Santa Cruz Biotechnology, Dallas, TX, USA; 1:200), NF-κB p65 (Cusabio, Wuhan, China; 1:1,000), IκB-α (Abcam; 1:5,000), phosphorylated-IκB-α (Abcam; 1:1,000), COX2 (Proteintech, Rosemont, IL, USA; 1:300), caspase-3 (Proteintech; 1:1,000), Bax (Proteintech; 1:2,000), Bcl2 (Cell Signaling Technology, Danvers, MA, USA; 1:1,000), histone 3 (Cell Signaling Technology; 1:1,000), and β-actin (Servicebio; 1:2,000). They were then incubated with HRP-conjugated secondary antibodies (Proteintech; 1:10,000). The Western blots were developed using a ChemiDoc MP Imaging System (Bio-Rad, Hercules, CA, USA); band intensities were quantified with the aid of Quantity One ver. 4.6.7 software (Bio-Rad).

### Immunofluorescence Staining

Cultured cells were incubated with anti-NF-κB p65 (Cusabio, 1:200) antibodies at 4°C overnight, then with CY3-labeled secondary antibodies (Servicebio; 1:500) for 1 h at room temperature and counterstained with 4′,6-diamidino-2-phenylindole (DAPI; Servicebio) for 10 min. Images were taken under a fluorescence microscope (Olympus Corp., Tokyo, Japan).

### Terminal Deoxynucleotidyl Transferase-Mediated Nick-End Labeling (TUNEL) Assay

We used the TUNEL assay to detect apoptotic cells in kidney sections; we employed an *in situ* Cell Death Detection Kit (Roche, Basel, Switzerland) following the manufacturer’s instructions. After deparaffinization and rehydration, kidney tissues were incubated with proteinase K (20 µg/ml) for 20 min at room temperature and with the TUNEL reaction mixture for 1 h at 37°C, stained with DAPI, and observed under a fluorescence microscope.

### Cytometric Bead Array (CBA)

A CBA Mouse Th1/Th2/Th17 Cytokine Kit (Becton Dickinson, Franklin Lakes, NJ, USA) was used to measure serum TNF-α and IL-6 levels. Standards were reconstituted in 2.0-ml amounts of assay diluent and serially diluted to 1:2, 1:4, 1:8, 1:16, 1:32, 1:64, 1:128, and 1:256; mixed capture beads (10 µl volumes) were added to all tubes, followed by 50-µl amounts of standard dilutions and sera, and then 50-µl amounts of the Th1/Th2/Th17 PE detection reagent. The tubes were incubated for 2 h at room temperature; next, 300 µl of wash buffer was added to each tube to resuspend the beads, and the beads were subjected to flow cytometry. Data were analyzed with the aid of FCAP Array Software.

### Statistical Analysis

All data are expressed as means ± standard deviations (SDs). Student’s *t*-test was used to compare pairs of groups; a one-way ANOVA followed by Tukey’s test was employed to compare multiple groups. We used SPSS ver. 19 software (IBM Corp., Armonk, NY, USA). A P-value <0.05 was taken to indicate statistical significance.

## Results

### Neferine Attenuated AKI Induced by I/R and LPS

**Figure 2:**
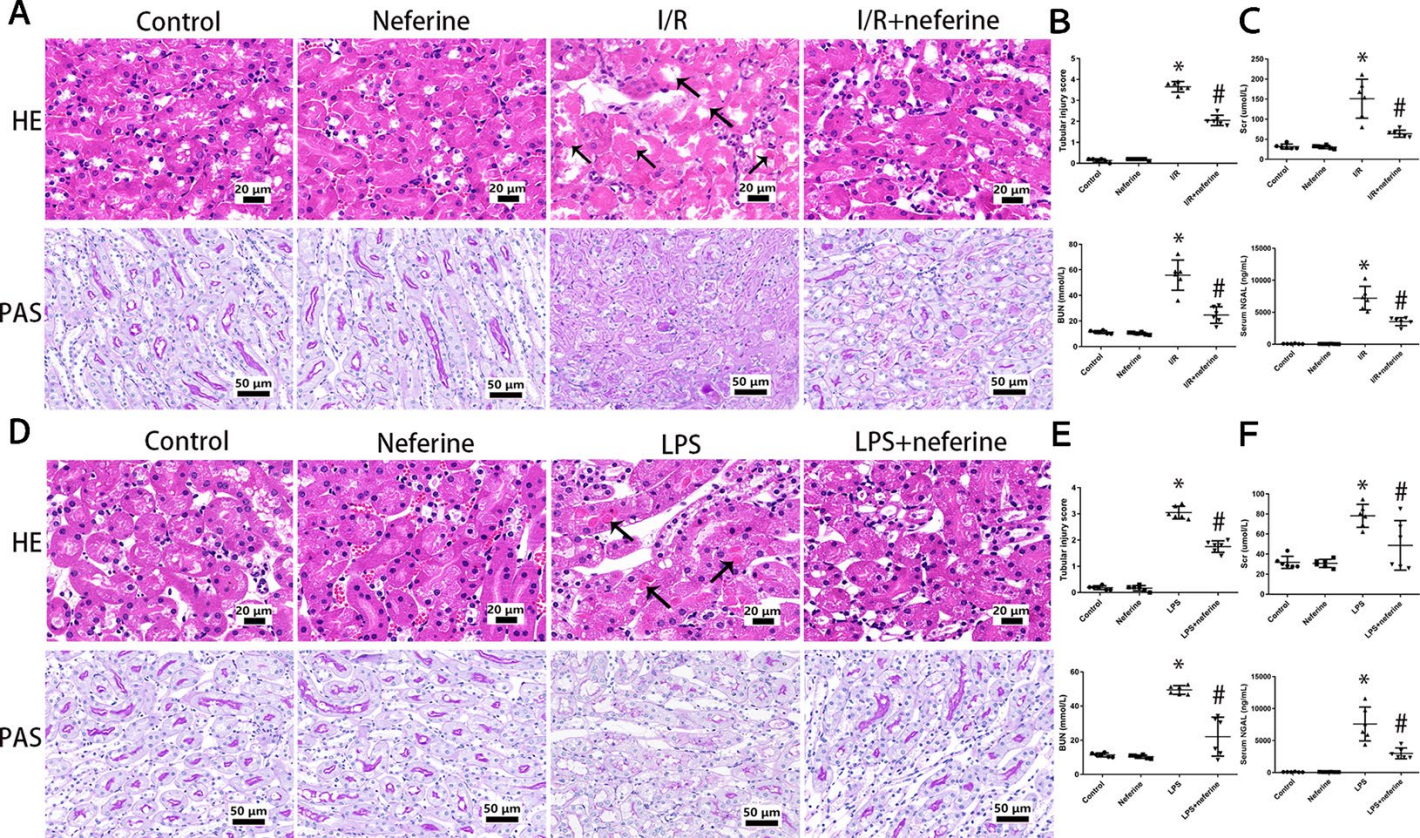
Neferine attenuated AKI induced by I/R and LPS *in vivo*. **(A**, **D)** Representative photomicrographs of kidney sections stained by hematoxylin–eosin and periodic acid–Schiff. Black arrows indicated tubular injury, comprising cast deposition, brush border loss, necrosis, tubular dilatation, and vacuolization. **(B**, **E)** The tubular injury scores of **(A** and **D)**. Data was presented as the percentage of damaged renal tubules over the total visible tubules in the outer medulla. **(C**, **F)** Serum levels of Scr, BUN, and NGAL in four subgroups. Data are represented as means ± SD (n = 6). *P < 0.05 *vs*. control, ^#^P < 0.05 *vs*. I/R or LPS.

In mice with AKI induced by I/R, the renal tubular epithelium was severely injured; the brush border was lost, and necrosis, cast deposition, and renal tubule dilatation were evident (**Figure 2A**). In the LPS-induced AKI model, tubular cell damage included vacuolization, brush border loss, cast deposition, and necrosis (**Figure 2D**). These pathological changes were most obvious in the external medulla. We calculated tubular injury scores (**Figures 2B, E**). No mesangial proliferation and fibrosis were observed in mouse AKI models. Renal insufficiency (reflected by increases in serum SCr, BUN, and NGAL levels) was evident in both AKI models (**Figures 2C, F**). Neferine pretreatment effectively mitigated kidney injury and renal insufficiency in both AKI models.

### Neferine Suppressed Apoptosis in Mouse AKI Models

**Figure 3:**
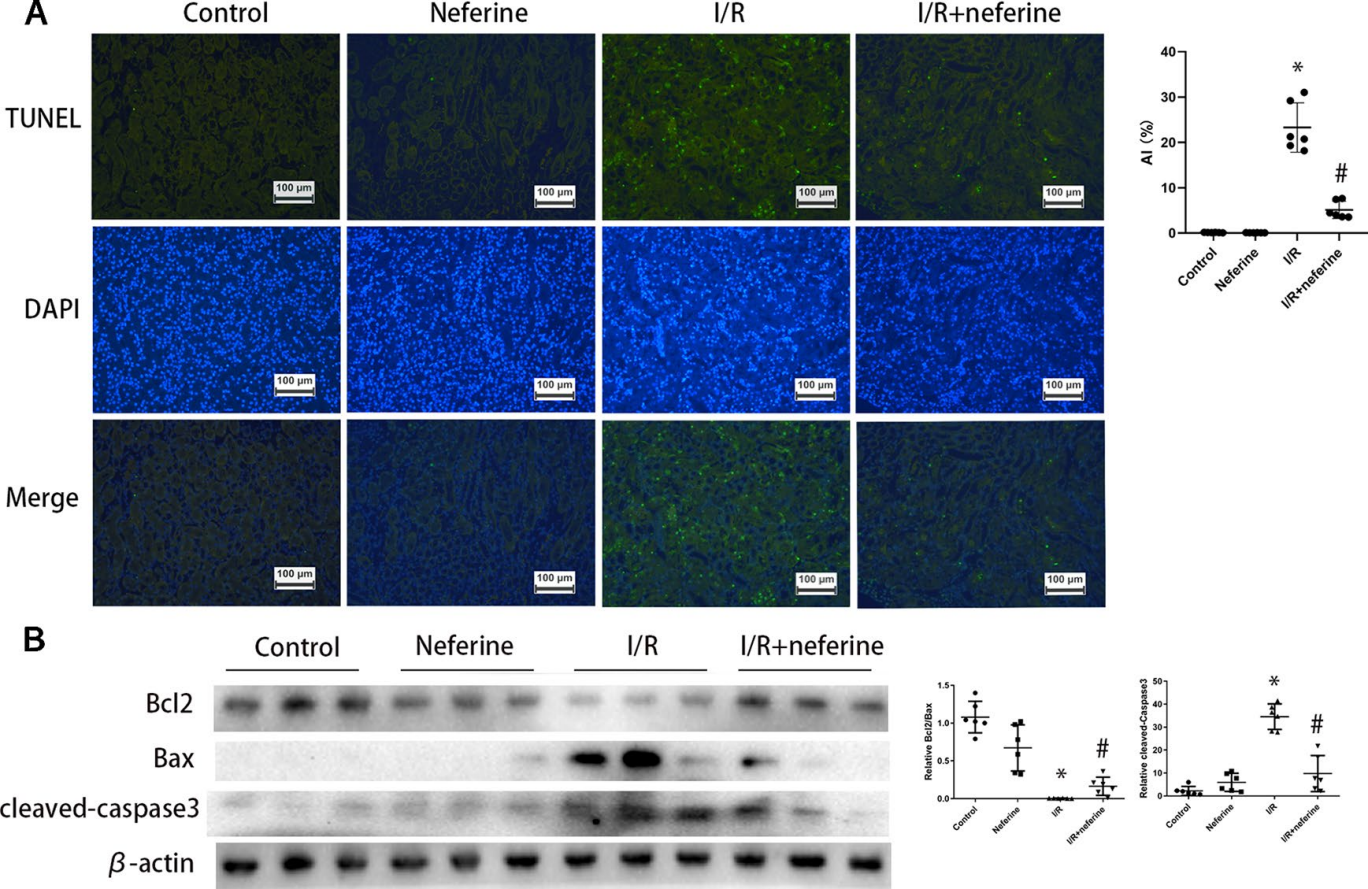
Neferine suppressed apoptosis in I/R-AKI. **(A)** Apoptosis induced by I/R in kidney cells was detected by TUNEL assays. Apoptosis index was calculated according to TUNEL results. **(B)** Renal protein levels of Bcl2, Bax, and cleaved caspase-3 assayed by Western blotting (three randomly samples selected from each subgroup were shown). The corresponding quantifications were shown as well. Data are represented as means ± SD (n = 6). *P < 0.05 *vs*. control, ^#^P < 0.05 *vs*. I/R.

**Figure 4:**
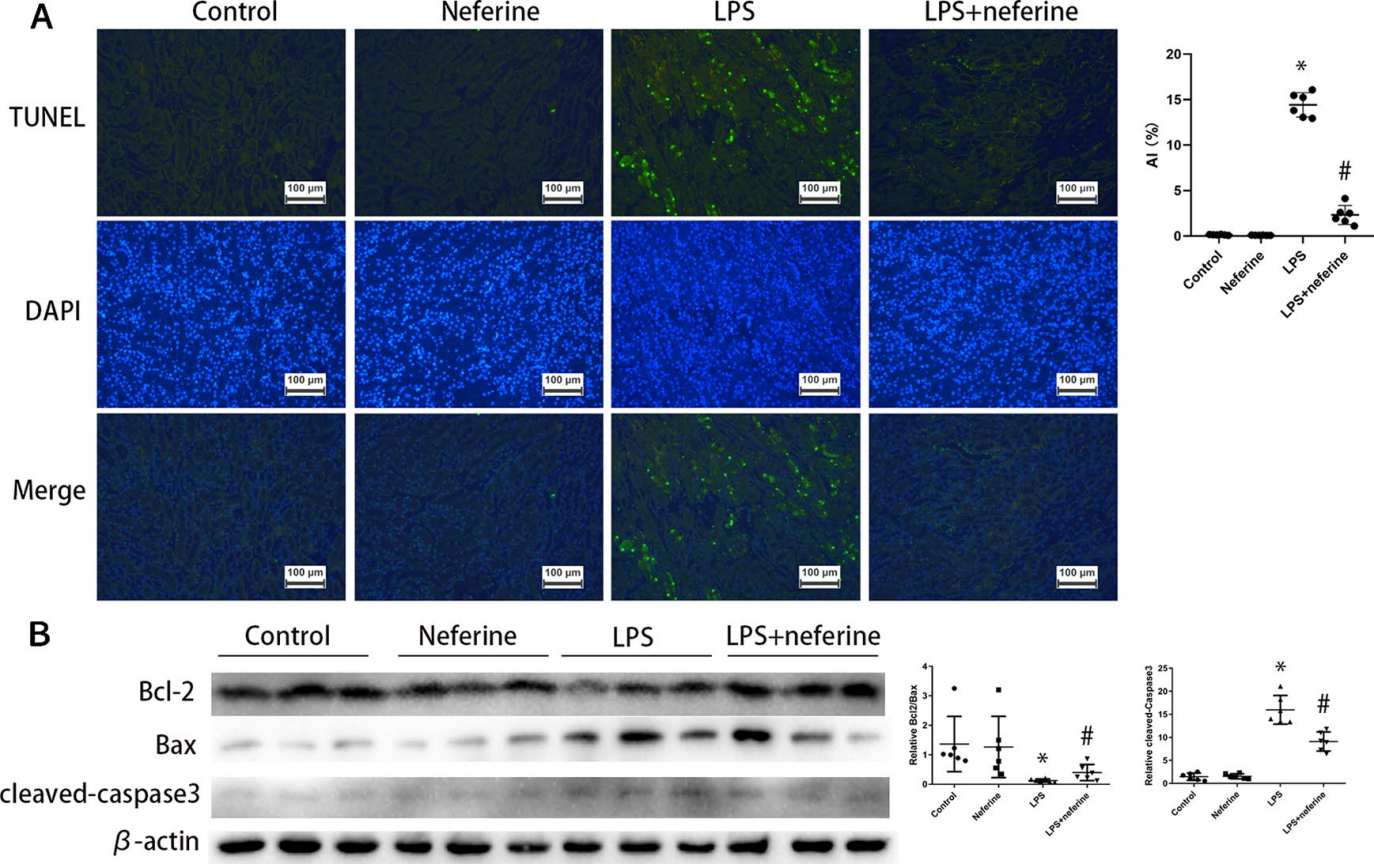
Neferine suppressed apoptosis in LPS-AKI. **(A)** Apoptosis induced by LPS in kidney cells was detected by TUNEL assays. Apoptosis index was calculated according to TUNEL results. **(B)** Renal protein levels of Bcl2, Bax, and cleaved caspase-3 assayed by Western blotting (three randomly samples selected from each subgroup were shown). The corresponding quantifications were shown as well. Data are represented as means ± SD (n = 6). *P < 0.05 *vs*. control, ^#^P < 0.05 *vs*. LPS.

Above, we showed that neferine afforded renal protection; we thus explored whether the drug affected apoptosis. Apoptosis was in play in both AKI mouse models as revealed by the TUNEL assay which detects DNA fragmentation (**Figures 3A** and **4A**). We further verified the apoptotic phenotype by showing that Bcl2 expression was decreased, while the Bax and activated caspase-3 levels were increased in model groups, compared to the control values (**Figures 3B** and **4B**). Neferine pretreatment significantly reduced DNA breakage and the changes in the expression levels of Bcl2, Bax, and cleaved caspase-3 induced by I/R and LPS (**Figures 3** and **4**).

### Neferine Attenuated the Expression of Inflammatory Cytokines in Mouse AKI Models

**Figure 5:**
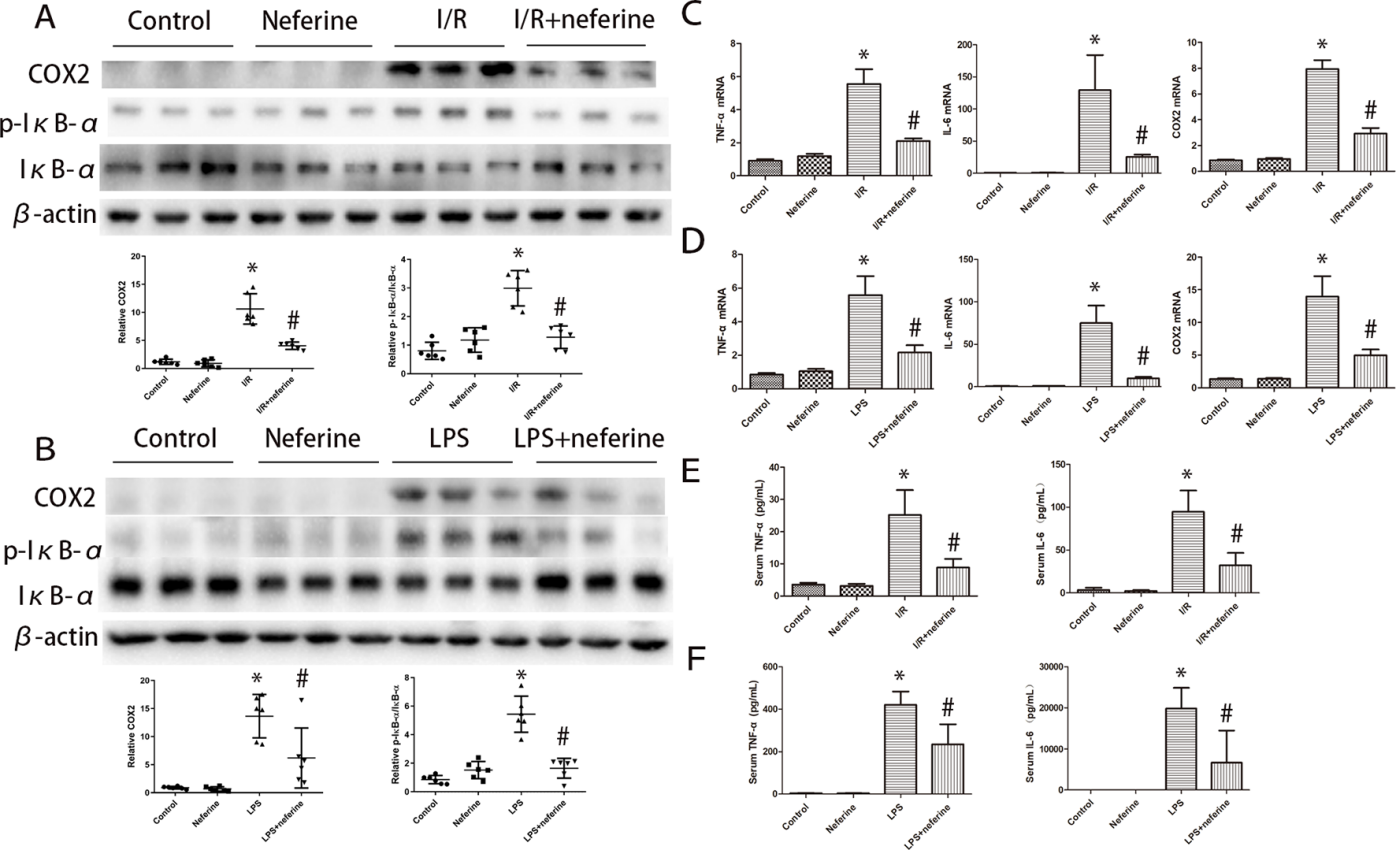
Neferine inhibited the phosphorylation of IκB-α and the expression of inflammation cytokines in AKI models. **(A**, **B)** Renal protein levels of COX2, p-IκB-α, and IκB-α assayed by Western blotting (three randomly samples selected from each subgroup were shown). The corresponding quantifications were shown as well. **(C**, **D)** Renal mRNA levels of TNFα, IL-6, and COX2 were assayed by qRT-PCR and quantified as fold changes. **(E**, **F)** Serum levels of TNFα and IL-6 in different subgroups. Data are represented as means ± SD (n = 6). *P < 0.05 *vs*. control, ^#^P < 0.05 *vs*. I/R or LPS.

Inflammation can be triggered by various stimuli, including I/R and LPS. Inflammatory factors play important roles in AKI emergence and development. The levels of mRNAs encoding TNF-α, IL-6, and COX2 in the kidney increased significantly 24 h after I/R or LPS injection (**Figures 5C, D**), as did the serum levels of TNF-α and IL-6 (**Figures 5E, F**) and the kidney COX2 level (as revealed by immunoblotting; **Figures 5A, B**). Neferine pretreatment effectively mitigated the abnormal expression of inflammatory mediators.

### Neferine Inhibited the Infiltration of Inflammatory Cells in Models of AKI

**Figure 6:**
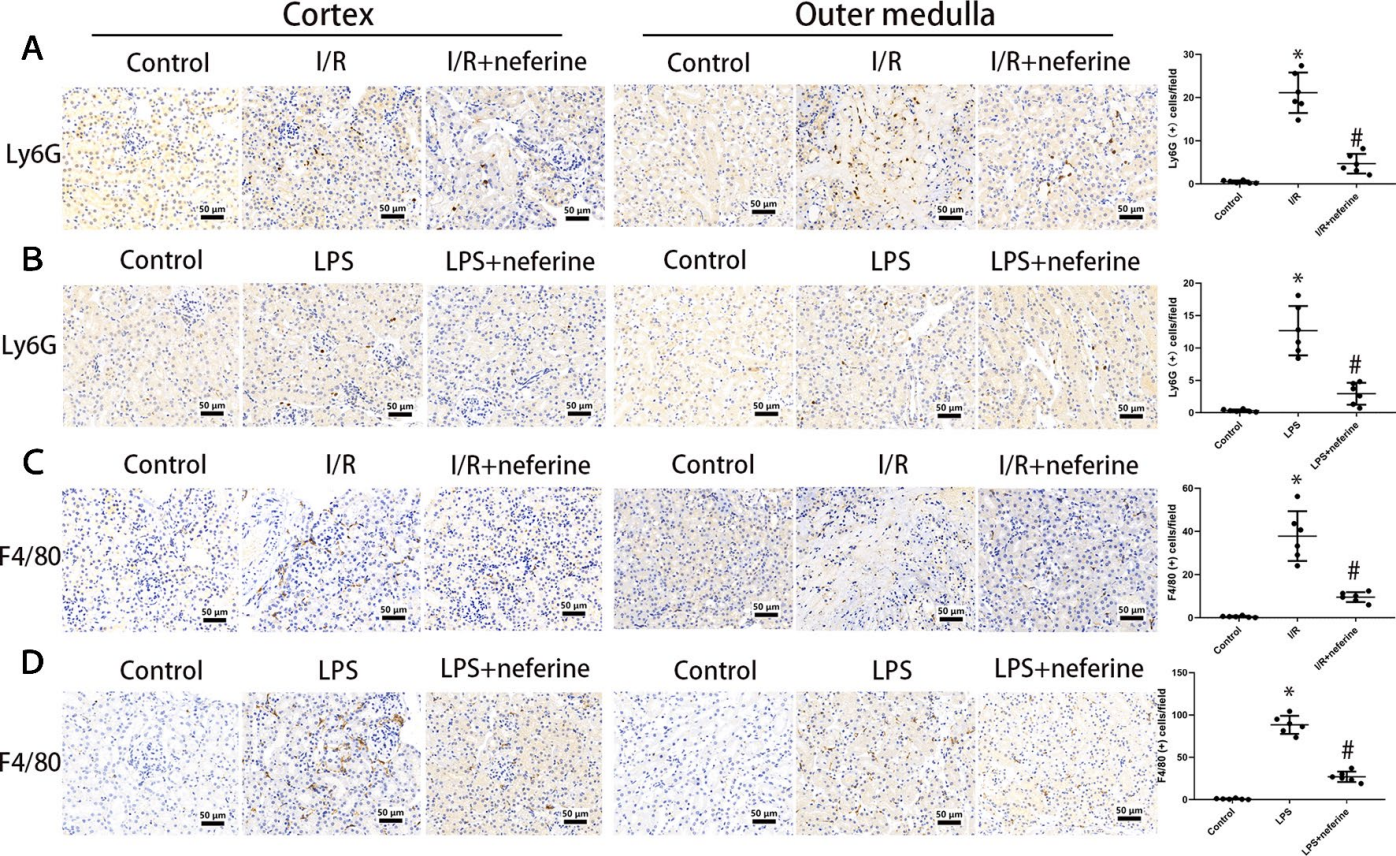
Neferine inhibited the infiltration of inflammatory cells in AKI models. The figures in cortex and outer medulla were listed respectively. **(A**, **B)** Photomicrographs of Ly6G-stained kidney sections and the corresponding quantifications. **(C**, **D)** Photomicrographs of F4/80-stained kidney sections and the corresponding quantifications. Data are represented as means ± SD (n = 6). *P < 0.05 *vs*. control, ^#^P < 0.05 *vs*. I/R or LPS.

The various leukocyte subgroups contribute to AKI in different ways. Upon the development of AKI, granulocytes and macrophages successively accumulate in kidney tissue (Bonventre and Zuk, 2004; Joannes-Boyau et al., 2010). We performed immunohistochemical staining for Ly6G and F4/80 as markers of granulocytes and macrophages, respectively. As shown in **Figures 6A–D**, granulocyte and macrophage infiltrations were negligible in the control and neferine subgroups. However, the expressions of Ly6G and F4/80 in both cortex and outer medulla were increased by I/R or administration of LPS. Neferine reduced the number of Ly6G- and F4/80-positive cells, suggesting inhibition of inflammatory cells infiltration.

### Neferine Inhibited the Nuclear Translocation of NF-κB in Both AKI Mouse Models

**Figure 7:**
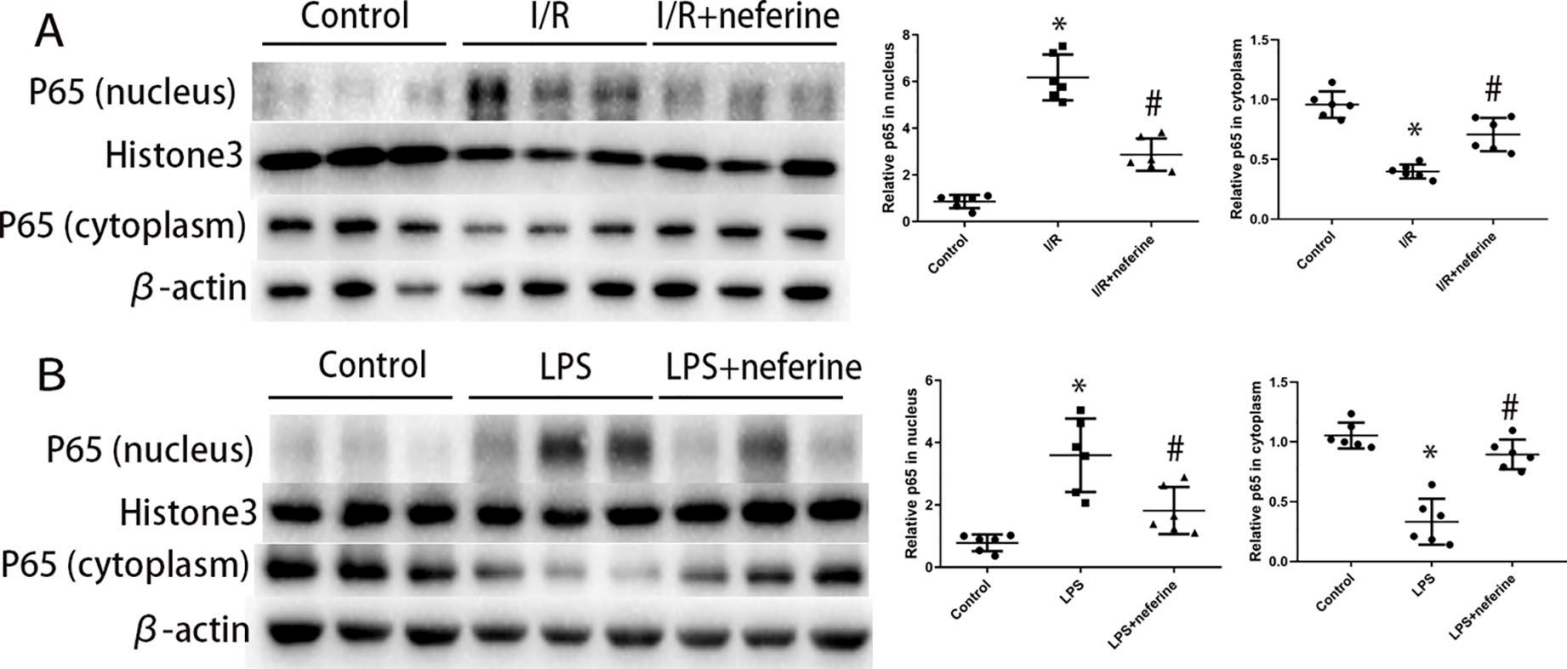
Neferine inhibited the nuclear translocation of NF-κB in AKI models. **(A**, **B)** P65 levels in nuclear extract and cytoplasm assayed by Western blotting (three randomly samples selected from each subgroup were shown). The corresponding quantifications were shown. Data are represented as means ± SD (n = 6). *P < 0.05 *vs*. control, ^#^P < 0.05 *vs*. I/R or LPS.

NF-κB activation is triggered by both I/R and LPS. NF-κB is activated principally *via* nuclear translocation. To explore whether neferine modulated NF-κB activity, we used Western blotting to measure the levels of NF-κB p65 in the nucleus and cytoplasm. The immunoblotting of p65 in nucleus and cytoplasm (**Figures 7A, B**) confirmed that neferine inhibited the nuclear translocation of NF-κB. Neferine reduced IκB-α phosphorylation and degradation after I/R or LPS injection (**Figures 5A, B**), which further revealed that neferine inhibited the activation of NF-κB pathway.

### Effect of Neferine on the Expression Levels of Klotho in AKI Models

**Figure 8:**
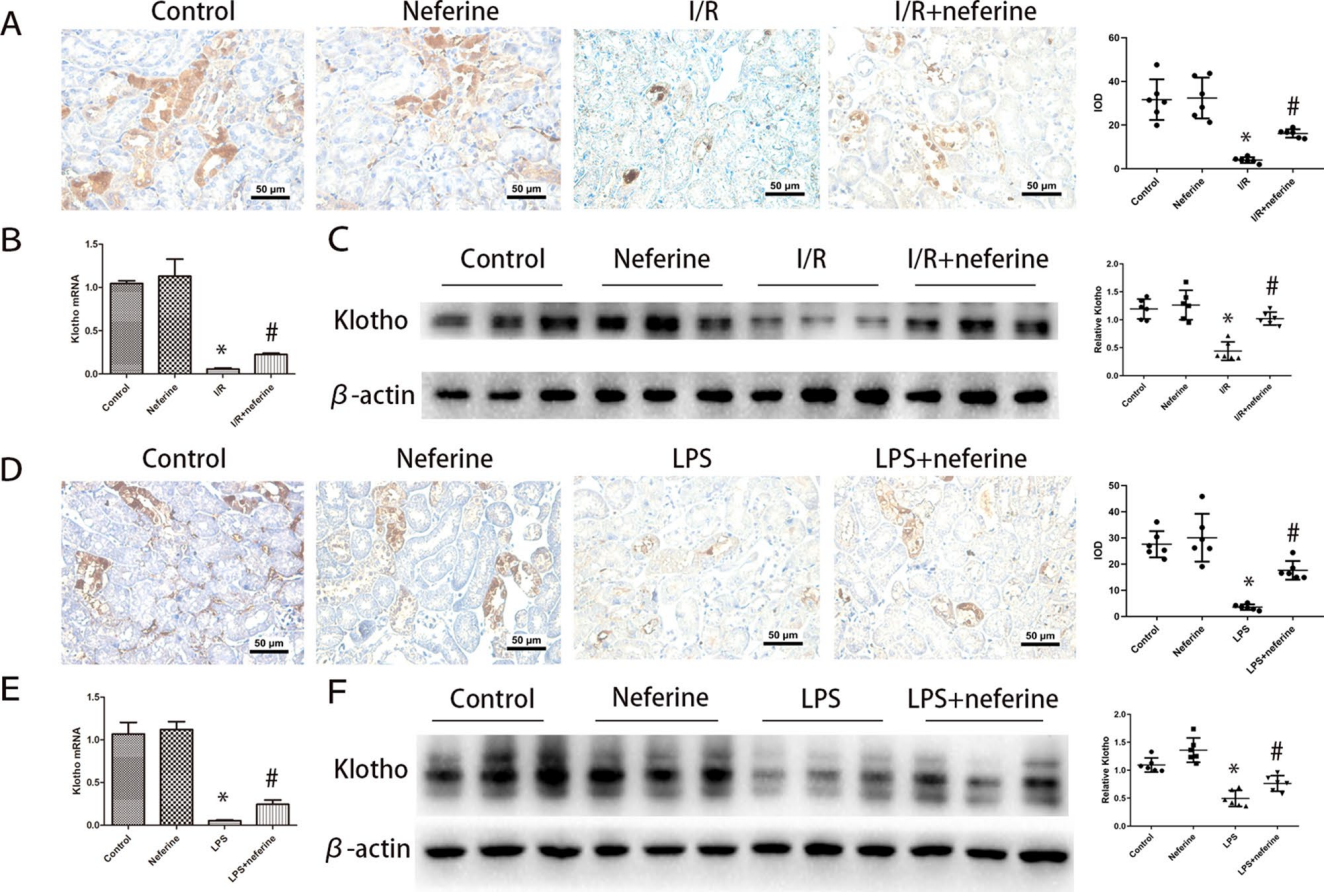
Effect of neferine on the expression levels of Klotho in AKI models. **(A**, **D)** Representative expression levels of Klotho by immunohistochemistry in different groups and the corresponding quantifications. **(B**, **E)** Renal mRNA levels of Klotho was assayed by qRT-PCR and quantified as fold changes. **(C**, **F)** Renal protein levels of Klotho assayed by Western blotting (three randomly samples selected from each subgroup were shown). The corresponding quantifications were shown as well. Data are represented as means ± SD (n = 6). *P < 0.05 *vs*. control, ^#^P < 0.05 *vs*. I/R or LPS.

We confirmed that both I/R (Panah et al., 2018) and LPS (Bi et al., 2018) downregulated Klotho expression. As mentioned above, Klotho levels decreased 3 h after I/R and attained a nadir 24 h after reperfusion (Hu et al., 2010). We thus performed assays at this time. The levels of Klotho-encoding mRNA and the protein *per se* were high in normal kidney tissues; both I/R (**Figures 8A–C**) and LPS (**Figures 8D–F**) triggered Klotho deficiencies. Neferine pretreatment attenuated such changes in both AKI models.

### Effect of Neferine on Apoptosis of NRK-52E Cells Exposed to H/R and LPS

**Figure 9:**
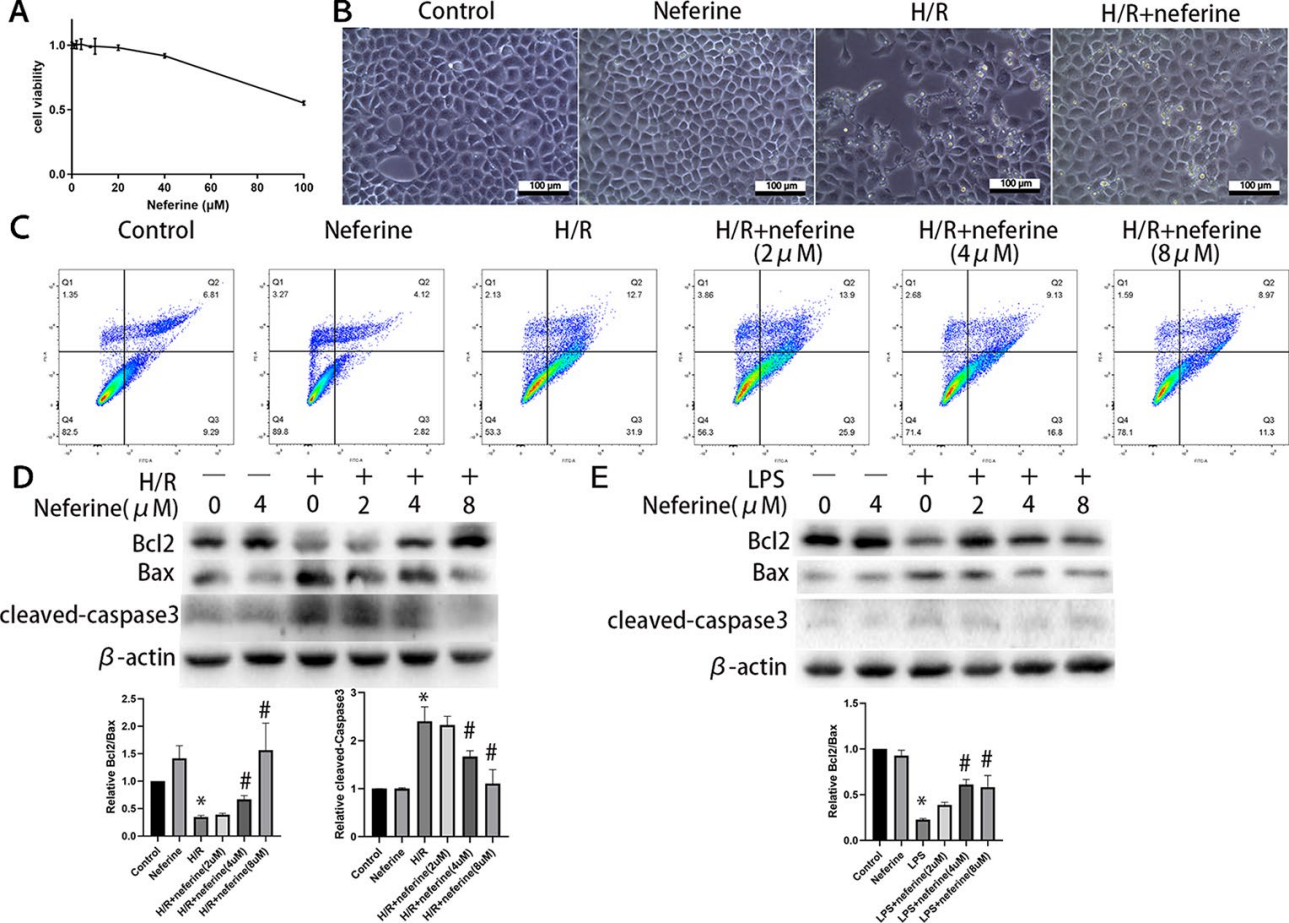
Neferine suppressed apoptosis in NRK-52E cells exposed to H/R or LPS. **(A)** Cell viability was measured by CCK8 in NRK-52E cells after 24 h of treatment with neferine. **(B)** The microscope image of NRK-52E cells. Cells were treated with H/R in the absence or presence of neferine (8 µM) for 12 h. **(C)** Cell apoptosis was detected by flow cytometry. NRK-52E cells were treated with H/R in the absence or presence of neferine (2, 4, 8 µM) for 12 h. **(D**, **E)** Western blotting of Bcl2, Bax, and cleaved caspase3. NRK-52E cells were treated with H/R or LPS in the absence or presence of neferine (2, 4, 8 µM) for 12 h. Each experiment was conducted independently three times. *P < 0.05 *vs*. control, ^#^P < 0.05 *vs*. H/R or LPS.

The above results suggest that neferine protects against AKI in mouse models in a manner involving tubular cells. Next, we investigated the mechanism of protection *in vitro* using NRK-52E cells. To determine the optimum concentration of neferine, a CCK8 assay of cell viability was performed using 1, 2, 4, 8, 10, 20, 40, and 100 μM neferine (**Figure 9A**), then neferine treatment with 2, 4, and 8 μM was employed in the following experiments. The morphological characteristics and the rate of apoptosis determined by flow cytometry (**Figures 9B, C**) suggested that neferine rescued the cell death induced by H/R. Moreover, Western blotting for Bcl2, Bax, and cleaved-caspase3 (**Figure 9D**) confirmed that neferine suppressed apoptosis. Although only a few apoptotic cells was observed after 24 h of LPS administration, the expression of Bcl2 and Bax protein levels revealed the protective effect of neferine in LPS-induced tubular cell injury (**Figure 9E**).

### Neferine Inhibits the Production of Inflammatory Cytokines and the Activation of NF-κB Pathway Induced by H/R and LPS in NRK-52E Cells

**Figure 10:**
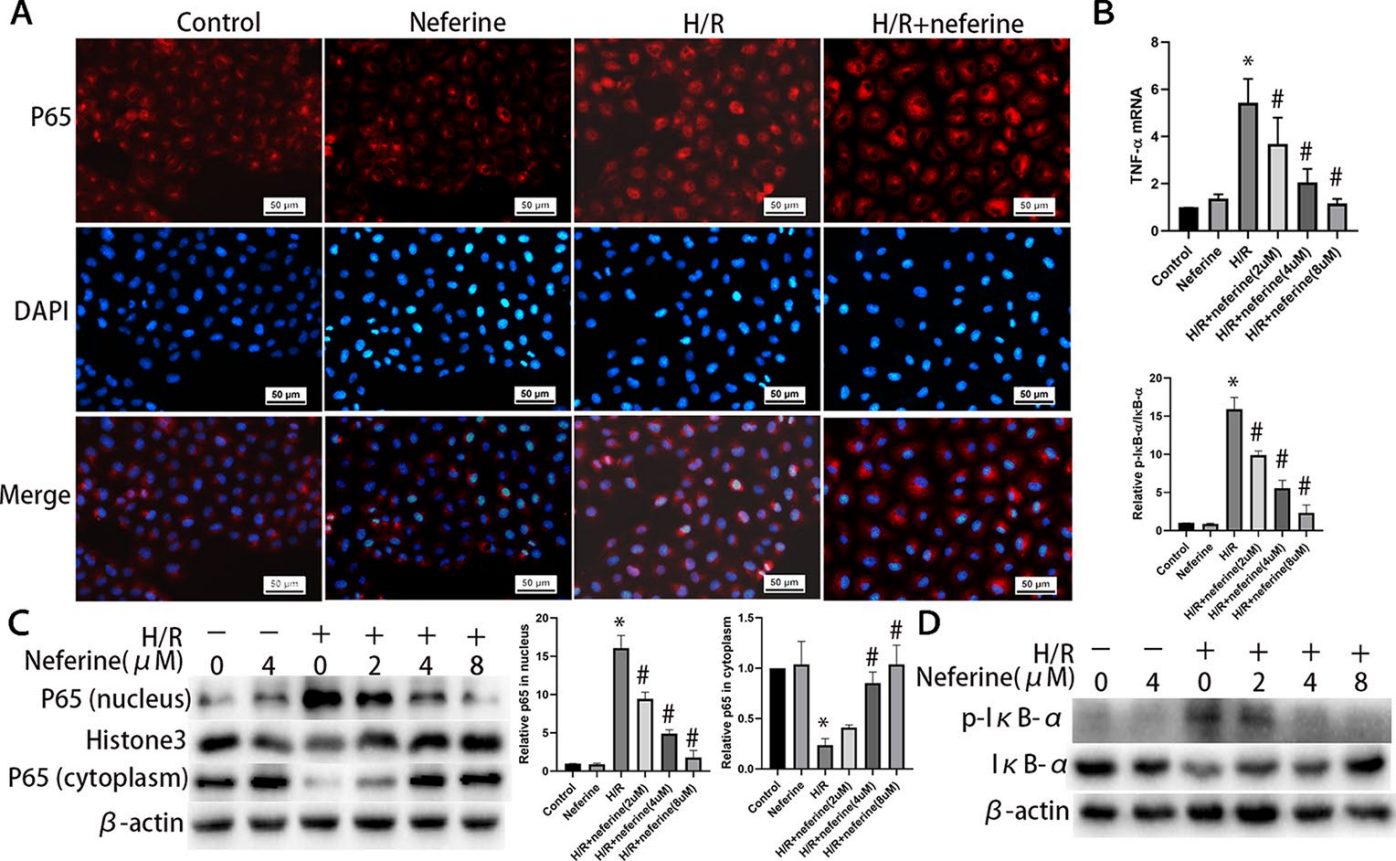
Neferine inhibited NF-κB signaling pathway and the production of inflammatory cytokine induced by H/R in NRK-52E cells. **(A)** Immunofluorescent staining of p65. NRK-52E cells were treated with H/R in the absence or presence of neferine (8 µM) for 12 h. The cells were stained with anti-p65 antibody (top panel) and counter-stained with DAPI (middle panel), and figures were merged (lower panel). **(B)** The TNF-α mRNA levels of NRK-52E cells assayed by qRT-PCR and quantified as fold changes. Cells were treated with H/R in the absence or presence of neferine (2, 4, 8 µM) for 12 h. **(C)** Western blotting of p65 in nucleus and cytoplasm. The corresponding quantifications were presented as well. **(D)** Western blotting of p-IκB-α and IκB-α in NRK-52E cells. Each experiment was conducted independently three times. *P < 0.05 *vs*. control, ^#^P < 0.05 *vs*. H/R.

**Figure 11:**
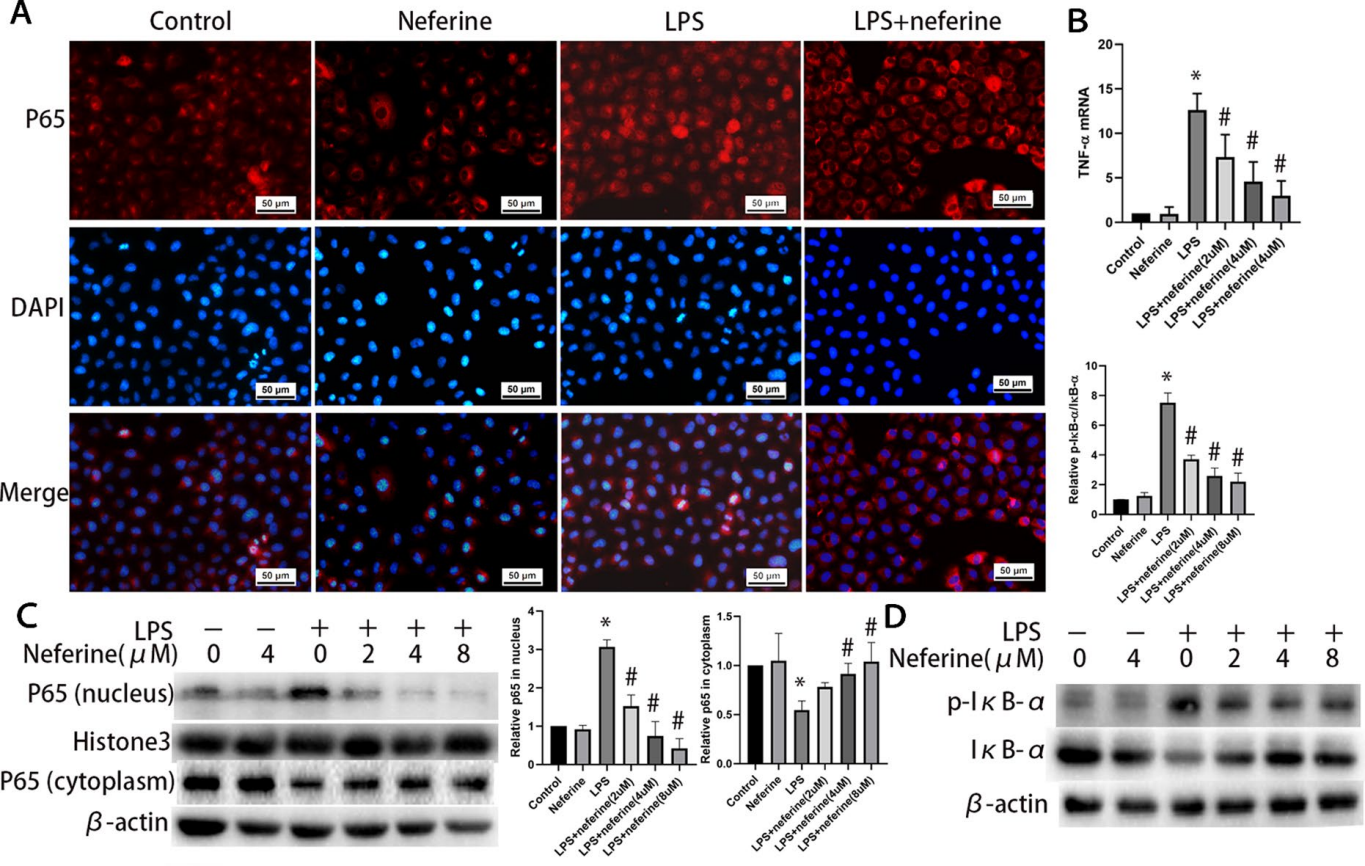
Neferine inhibited NF-κB signaling pathway and the production of inflammatory cytokine induced by LPS in NRK-52E cells. **(A)** Immunofluorescent staining of p65. NRK-52E cells were treated with LPS in the absence or presence of neferine (8 µM) for 12 h. The cells were stained with anti-p65 antibody (top panel) and counter-stained with DAPI (middle panel), and figures were merged (lower panel). **(B)** The TNF-α mRNA levels of NRK-52E cells assayed by qRT-PCR and quantified as fold changes. Cells were treated with LPS in the absence or presence of neferine (2, 4, 8 µM) for 12 h. **(C)** Western blotting of p65 in nucleus and cytoplasm. The corresponding quantifications were presented as well. **(D)** Western blotting of p-IκB-α and IκB-α in NRK-52E cells. Each experiment was conducted independently three times. *P < 0.05 *vs*. control, ^#^P < 0.05 *vs*. LPS.

Neferine dose-dependently inhibited the expression of TNF-α induced by H/R and LPS (**Figures 10B** and **11B**). Neferine inhibits the NF-κB signaling pathway *in vivo*; therefore, we next investigated the underlying mechanisms *in vitro*. Immunofluorescence staining (**Figures 10A** and **11A**) and Western blotting (**Figures 10C** and **11C**) showed the protein levels of p65 in the nucleus and cytoplasm, suggesting that neferine suppressed the nuclear translocation of NF-κB in a dose-dependent manner. Also, neferine reduced the phosphorylation and degradation of IκB-α induced by H/R and LPS (**Figures 10D** and **11D**), consistent with the *in vivo* findings.

### Effect of Neferine on Klotho Expression in NRK-52E Cells

**Figure 12:**
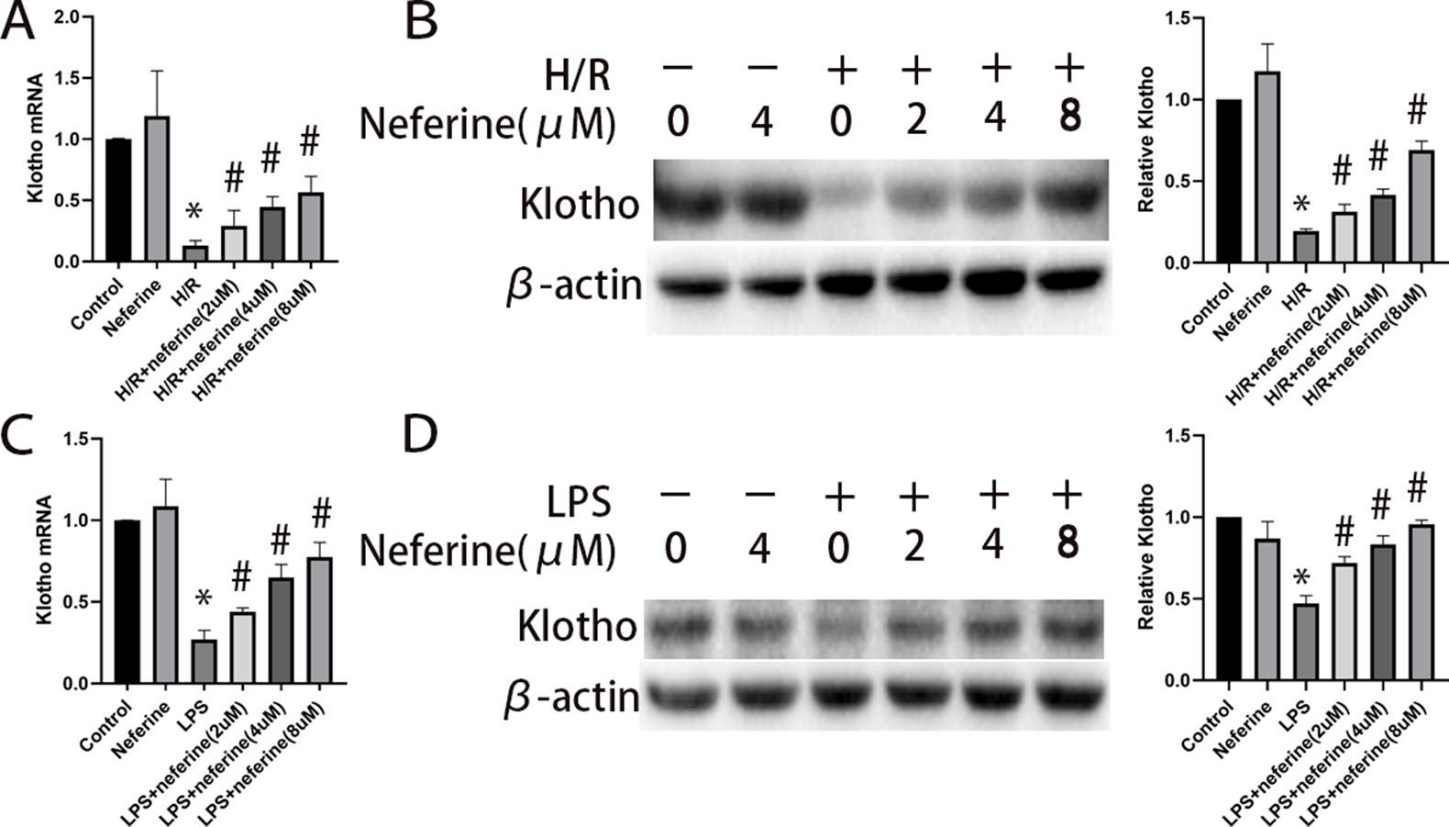
Effect of neferine on the expression levels of Klotho in NRK-52E cells exposed to H/R or LPS. Cells were treated with H/R or LPS in the absence or presence of neferine (2, 4, 8 µM) for 12 h. **(A**, **C)** The Klotho mRNA levels of NRK-52E cells assayed by qRT-PCR and quantified as fold changes. **(B**, **D)** Protein levels of Klotho assayed by Western blotting and the corresponding quantifications. Each experiment was conducted independently three times. *P < 0.05 *vs*. control, ^#^P < 0.05 *vs*. H/R or LPS.

The gene that encodes Klotho is expressed mainly in distal renal tubules, while Klotho protein is present in proximal renal tubules. We investigated the expression of the Klotho-encoding gene using NRK-52E cells (Hsu et al., 2014a). The expression level of Klotho in NRK-52E cells was reduced by exposure to H/R and LPS. Administration of neferine significantly increased the Klotho mRNA and protein levels in a dose-dependent manner (**Figures 12A–D**).

## Discussion

A multicenter, retrospective Chinese cohort study (Xu et al., 2015) found that the overall incidence of AKI was 11.6% and in-hospital mortality 8.8%. AKI is thus a major problem; no effective drug is available, and such a drug is urgently required. We showed that neferine, a bisbenzylisoquinoline alkaloid, effectively alleviated AKI by inhibiting apoptosis, inflammation, and nuclear translocation of NF-κB while preserving Klotho expression.

AKI caused by I/R and sepsis develops secondarily to several clinical conditions. We explored this topic using I/R and LPS AKI models, which were reliable and reproducible. AKI is characterized by kidney apoptosis and inflammation; a drug ameliorating these symptoms would be very useful. Neferine was renoprotective in AKI mouse models, as evidenced both pathologically and biochemically. Neferine accumulates to high levels in the kidneys, perhaps explaining the beneficial effects afforded by the drug (Huang et al., 2007). Neferine highly penetrates tissues by passive diffusion due to its highly lipophilicity. The drug then plays various pharmacological roles. This is the first report to discover the potential therapeutic value of neferine for AKI.

In I/R-AKI, proximal tubule epithelial cells are extremely susceptible to apoptosis, and loss of tubular cells causes increased backleak and reduced glomerular filtration rate (GFR) (Havasi and Borkan, 2011). Although the pathogenesis of septic AKI is multifactorial, renal cell apoptosis is definitely involved (Joannes-Boyau et al., 2010). In our study, neferine decreased the ratio of Bax/Bcl2 *in vivo* and *in vitro* and dramatically attenuated the apoptosis of tubular cells in AKI, which suggested that anti-apoptosis may be one of the mechanisms of kidney protection from neferine. Pyroptosis is a new cell death pathway drawing substantial attention in recent years (Yang et al., 2014). It was reported that neferine inhibited LPS-ATP-induced endothelial cell pyroptosis (Tang et al., 2019), indicating the effect of prohibiting pyroptosis of neferine. Therefore, further studies focused on pyroptosis should be evaluated to explore the functional mechanism of neferine in AKI.

The NF-κB family of transcription factors integrates extracellular stimuli with intracellular signal transduction pathways, transcriptionally regulating genes involved in inflammation, immunity, and apoptosis. NF-κB is a potential target of anti-inflammatory therapy. Various etiologies of AKI, including I/R and LPS, can trigger the activation of NF-κB through distinct pathway in tubular cells (Sanz et al., 2010; Panah et al., 2018). The classic activation pathway features three consecutive steps: phosphorylation/ubiquitination, proteolytic degradation of IκB, and NF-κB nuclear translocation. As most NF-κB target genes are regulated by RelA (p65)/p50 (Sanz et al., 2010), we measured p65 levels when exploring the effects of NF-κB. We found that neferine clearly inhibited NF-κB nuclear translocation, indicating that suppression of NF-κB activation may explain the renoprotective action of the drug. Neferine reduced IκB-α phosphorylation and degradation, suggesting that the drug affected the classical NF-κB activation pathway. The results were consistent with the conclusions in osteoclast differentiation (Chen et al., 2019). Moreover, unlike other types of cells, neferine also suppressed NF-κB pathway by inhibiting the p65 expression in renal cancer cells (Kim et al., 2019).

TNF-α, IL-6, and COX2, all of which are involved in the AKI inflammatory response (Lemay et al., 2000; Grigoryev et al., 2008; Feitoza et al., 2010; Wang et al., 2016), are downstream regulators of NF-κB (Baskaran et al., 2016). Expression of the genes encoding these proinflammatory proteins is upregulated after NF-κB enters the nucleus and therein binds to the corresponding promoters. We found that the serum levels of TNF-α, IL-6, and COX-2 expressions in the kidney increased substantially in both AKI models. Neferine pretreatment inhibited cytokine upregulation in AKI, accompanied by the inhibition of NF-κB activation. We thus suggest that neferine relieves AKI inflammation by preventing NF-κB nuclear translocation.

The Klotho protein comes in two different forms: transmembrane and soluble Klotho (sKL). Transmembrane Klotho serves as a co-receptor for fibroblast growth factor-23 (FGF23) and thus regulates both calcium and phosphorus metabolism. sKL, the extracellular domain of the transmembrane form of Klotho, protects the kidney by exerting anti-oxidative (Mitobe et al., 2005), anti-apoptosis (Hu et al., 2012), anti-inflammation (Oh et al., 2015; Bi et al., 2018), anti-fibrotic effects (Sugiura et al., 2012). Klotho expression was reduced after the induction of AKI by I/R, LPS, cisplatin (Panesso et al., 2014), and folic acid (Moreno et al., 2011). Low-level Klotho is both a biomarker of and a contributor to AKI pathogenesis. Klotho is upregulated by resveratrol (Hsu et al., 2014a), testosterone (Hsu et al., 2014b), statins (Kuwahara et al., 2008), Rhein (Zhang et al., 2017), and hydrogen-rich saline (Chen et al., 2017) in both the cells and tissues of animals with kidney disease. Klotho restoration exerted impressive renal protective roles on pathological injury. We found that the Klotho levels fell in AKI, consistent with previous work. Notably, the present study demonstrated that neferine significantly prevented Klotho loss from AKI, supporting the scenario that the renoprotective effect of neferine might be mediated by the upregulation of Klotho expression.

The relationship between the Klotho protein and NF-κB pathway activity is critical. TNF-α downregulates Klotho expression *via* an NF-κB-dependent mechanism in renal tubular cells (Moreno et al., 2011). Preservation of Klotho expression attenuates inflammation induced by LPS *via* inhibition of the TLR4/NF-κB pathway (Bi et al., 2018). We found that neferine not only inhibited NF-κB nuclear translocation but also reversed the decrease in Klotho expression. The function mechanisms of neferine were supposed to be one of the following three ways. Neferine may inhibit the NF-κB pathway directly. Alternatively, neferine may inhibit the NF-κB pathway by increasing Klotho expression. Finally, neferine may affect NF-κB activation and Klotho expression *via* two different signaling pathways. Further researches should be performed to explore the exact mechanisms.

In summary, we found that neferine protected against AKI by inhibiting both apoptosis and inflammation. The renoprotection afforded by the drug may be attributable to inhibition of NF-κB activation and preservation of Klotho expression. Neferine may be a valuable prophylactic therapy for AKI.

## Data Availability Statement

The raw data supporting the conclusions of this manuscript will be made available by the authors, without undue reservation, to any qualified researcher.

## Ethics Statement

The animal study was reviewed and approved by The Institutional Animal Care and Use Committee of Central South University.

## Author Contributions

Conceived and designed the experiments: HL and QZ. Performed the experiments: HL, YC and WC. Analyzed the data: HL and WC. Contributed reagents/materials/analysis tools: PX, JX and RT. Wrote the manuscript: HL and WC.

## Funding

This study was ﬁnancially supported by the National Youth Science Foundation of China (No. 81600536) and the Natural Science Foundation of Hunan Province of China (No. 2018JJ3833; No. 2018JJ3818).

## Conflict of Interest

The authors declare that the research was conducted in the absence of any commercial or financial relationships that could be construed as a potential conflict of interest.
